# Compression Behavior of 3D Printed Composite Isogrid Structures

**DOI:** 10.3390/polym16192747

**Published:** 2024-09-28

**Authors:** Marina Andreozzi, Carlo Bruni, Archimede Forcellese, Serena Gentili, Alessio Vita

**Affiliations:** Dipartimento di Ingegneria Industriale e Scienze Matematiche (DIISM), Università Politecnica delle Marche, Via Brecce Bianche 12, 60131 Ancona, Italy; c.bruni@staff.univpm.it (C.B.); a.forcellese@univpm.it (A.F.); s.gentili@pm.univpm.it (S.G.); alessio.vita@staff.univpm.it (A.V.)

**Keywords:** 3D printing, composites, isogrid

## Abstract

Composite materials, particularly carbon fiber-reinforced polymers (CFRPs), have become a cornerstone in industries requiring high-performance materials due to their exceptional mechanical properties, such as high strength-to-weight ratios, and their inherent lightweight nature. These attributes make CFRPs highly desirable in aerospace, automotive, and other advanced engineering applications. However, the compressive behavior of CFRP structures remains a challenge, primarily due to the material sensitivity to structural instability, leading to matrix cracking and premature failure under compressive loads. Isogrid structures, characterized by their unique geometric patterns, have shown promise in enhancing the compressive behavior of CFRP panels by providing additional support that mitigates these issues. Traditionally, these structures are manufactured using automated techniques like automated fiber placement (AFP) and automated tape laying (ATL), which, despite their efficacy, are often cost-prohibitive for small-scale or custom applications. Recent advancements in 3D-printing technology, particularly those involving continuous fiber reinforcement, present a cost-effective and flexible alternative for producing complex CFRP structures. This study investigates the compressive behavior of 3D-printed isogrid structures, fabricated using continuous carbon fiber reinforcement via an Anisoprint Composer A3 printer equipped with towpreg coextrusion technology. A total of eight isogrid panels with varying infill percentages were produced and subjected to buckling tests to assess their performance. The experimental results indicate a direct correlation between infill density and buckling resistance, with higher infill densities leading to increased buckling loads. Additionally, the failure modes were observed to shift from local to global buckling as the infill density increased, suggesting a more uniform distribution of compressive stresses. Post-test analyses using optical microscopy and scanning electron microscopy (SEM) revealed the presence of voids within the 3D-printed structures, which were found to negatively impact the mechanical performance of the isogrid panels. The findings of this study demonstrate that 3D-printed isogrid CFRP structures can achieve significant buckling resistance, making them a viable option for high-performance applications. However, the presence of voids remains a critical issue, highlighting the need for process optimizations in 3D-printing techniques to enhance the overall performance and reliability of these structures.

## 1. Introduction

Composite materials are well known both in the scientific literature and in several industries for their remarkable mechanical performances conjugated to their lightness. Their applications vary from aerospace to automotive industries, where material performances are predominant with respect to costs. Carbon fiber-reinforced polymers, based on continuous filaments, are the preferred type of composites, as the highest specific characteristics are required [1]. Indeed, this kind of material presents very high stiffness, tensile strength, and fracture resistance. However, concerning compression behavior, CFRPs demonstrate some limitations. This is related to the tendencies of CFRP structures to buckle and their reinforcement fibers to micro-buckle, both compromising structural integrity [2]. Moreover, the polymer matrix, in which the fibers are embedded, is prone to cracking and delamination under compressive loads, thus reducing the material resistance [3]. To mitigate this problem, isogrid structures can be used to reinforce thin panels and to provide more compression resistance without adding significant weight [4,5,6,7]. For this reason, isogrid-reinforced panels are used in demanding applications such as fuselage for aircraft [8]. Isogrids are lattice structures typically produced by automated fiber placement or automated tape laying (ATL) [9]. These two manufacturing methods, which are based on the use of robotic arms, are suitable for manufacturing components of very large dimensions, such as those used in aircraft and the energy sector [10].

The advent of 3D-printing technology, particularly for thermoplastic matrices with continuous reinforcement, has significantly enhanced the ability to fabricate intricate structures with high precision, notably for smaller components [11,12], especially for components with limited dimensions. CFRPs can be 3D printed using a process similar to the one used for printing plastic products, specifically the fused filament fabrication (FFF) method [13]. However, handling the continuous reinforcements in the extrusion process is challenging. Indeed, extruding continuous filaments presents several drawbacks. The first drawback is fraying, which can occur during filament movement. It happens when the fibers start to unravel or separate, thus compromising the composite integrity and aesthetic appearance [14]. Another problem typical of continuous reinforcement extrusion is related to filament sewing, a phase necessary to produce components without shape constraints. Cutting a filament could lead to fiber damage, binder removal, and limitations in adhering to the predefined path for fiber deposition. Another issue worth noticing is filament impregnation. Indeed, using high-viscosity matrices like thermoplastics can result in incorrect impregnations within the heated nozzle. Even though the nozzle reaches a temperature that significantly reduces the viscosity of the thermoplastic matrix, the materials’ rheology may prevent the thermoplastic from penetrating the fiber bundle. This results in voids within the 3D-printed composite, leading to a decrease in the mechanical performance [15].

Some examples of 3D-printed isogrid structures can be found in the scientific literature. Li et al. investigated the effect of geometric parameters on the compression strength of 3D-printed PVC isogrid, employing a numerical simulation to predict failure load [16]. Guajardo-Travino et al. investigated the effect of raster gaps and infill void in the compressive behavior of 3D-printed plastic isogrids [17]. However, all these studies were concerned with the use of unreinforced plastics for 3D printing isogrid structures. Some of the authors of these papers conducted research on isogrid structures 3D printed with short and discontinuous fibers. They studied the effect of reinforcing rib dimension and moisture absorption on the compressive behavior of isogrids produced in short carbon fiber-reinforced polyamide by an FFF process [18,19,20]. The studies found that varying the dimensions of the ribs could lead isogrids to fail under local or global buckling when subjected to compressive loads. In addition, they noted an improvement in compression resistance due to the structure drying after 3D printing.

Despite the widespread application of CFRPs, understanding the compressive behavior of 3D-printed CFRPs, particularly in isogrid configurations, remains limited. A notable gap exists in the scientific literature regarding the compression behavior of 3D-printed isogrid structures that utilize continuous reinforcement. Currently, two commercial desktop 3D printers, namely, the Markforged Mark Two and the Anisoprint Composer, are available for 3D printing with continuous CFRPs. The Anisoprint Composer in particular employs composite fiber co-extrusion (CFC) technology, which co-extrudes a composite material comprising a thermoplastic matrix and continuous fiber reinforcement [21]. To facilitate the handling of the continuous reinforcement, it is impregnated with a thermoset binder before the printing process [22]. As the fibrous reinforcement passes through the extrusion nozzle, it is impregnated with the thermoplastic matrix, resulting in a dual-matrix system. Anisoprint dual-matrix technology offers numerous advantages, including the ability to design and manufacture lightweight yet extremely strong components with improved tensile, flexural, and torsional strength. This technology also allows for the customization of fiber reinforcement within the matrix, optimizing the design to meet specific structural and functional requirements. The control over the distribution of fibers and matrix enables modulation of the stiffness and mechanical strength in specific directions, significantly enhancing the performance of the final components compared to traditional manufacturing methods. Also, this dual-matrix configuration provides unique mechanical properties, making it essential to explore and understand its behavior, especially under compressive loads in complex geometries like isogrid structures [23,24].

This paper aims to analyze the impact of different infill percentages on the compressive behavior of 3D-printed isogrid structures fabricated from continuous carbon fiber-reinforced polymers using Anisoprint dual-matrix technology. If adding more material to a structure by increasing the infill percentage leads to an increase in its overall strength, this does not always guarantee an improvement in specific strength, which is the strength relative to the material weight. Indeed, previous studies [16,20] have shown that increasing material usage can reduce specific strength, particularly if the added material induces a different failure mode. To analyze the effect of the infill percentage on the strength and specific strength of isogrids, eight distinct structures were produced, each with varying infill percentages. Compressive tests were conducted on standard specimens, while buckling tests were performed specifically on the isogrid panels. In addition, the isogrid structures were weighed prior to testing to calculate their specific compressive resistance. These results were also compared with those of short carbon fiber-reinforced structures to better understand the role continuous fibers play in influencing buckling behavior. Then, detailed analyses were carried out on the tested structures to determine whether global buckling of the overall structure or local buckling of the reinforcements occurred. Finally, the fractured surfaces of the tested samples were examined using scanning electron microscopy (SEM) and high-resolution three-dimensional tomography to investigate the internal structures and defects within the 3D-printed composites. 

## 2. Materials and Methods

### 2.1. Materials and 3D-Printing Process

**Figure 1:**
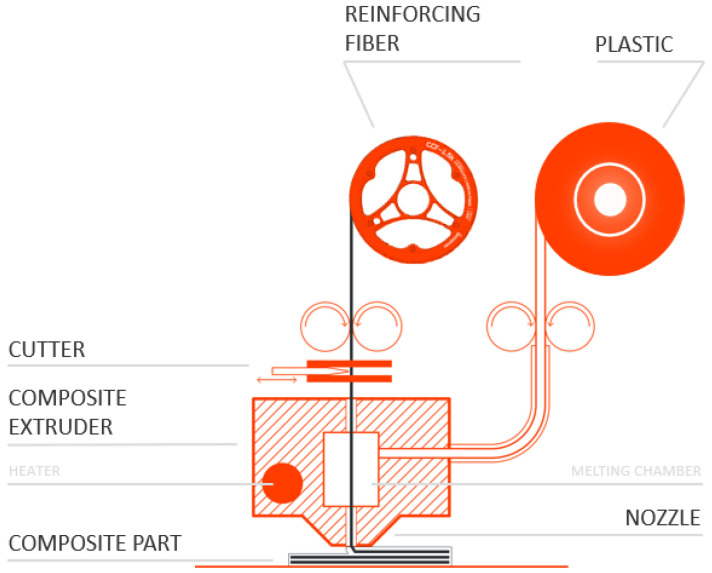
Scheme of the printing process of the Anisoprint A3 machine.

In the present study, isogrid structures were manufactured using fused filament fabrication technology based on CFC, which enables the printing of thermoplastic polymer matrix composites reinforced with continuous carbon fibers. The printing system employed was the Anisoprint Composer A3, developed by Anisoprint Inc., Esch-sur-Alzette, Luxembourg, as illustrated schematically in Figure 1. The machine’s print head consists of two extruders: one for extruding plastic material only (FFF) and the other for extruding composite material (CFC). Dual-matrix Anisoprint technology enables the simultaneous co-extrusion of a thermoplastic matrix and a bundle of thermoset resin-impregnated reinforcing fibers, allowing the fabrication of composite structures with optimized mechanical and physical properties. In the process under consideration, a bundle of epoxy resin-bonded carbon fibers and a thermoplastic filament are fed into a fusion chamber through two separate inlet tubes. Inside the chamber, the thermoplastic material is melted at a controlled temperature, allowing both the thermoset-impregnated fibers and the thermoplastic matrix to be extruded simultaneously through a single nozzle. This approach allows for the creation of a continuous dual-matrix composite structure, combining the advantages of the thermoplastic matrix, such as toughness and ease of melting, with the high mechanical properties of reinforced fibers impregnated with thermosetting resin.

CFC PA polyamide is a specialized thermoplastic developed by Polymaker for use with Anisoprint Technology in composite 3D printing. Its molecular structure of repeating amide bonds imparts notable mechanical strength and thermal stability. When melted, CFC PA’s viscosity decreases significantly, enhancing the penetration between reinforcing fiber bundles during printing, which results in composites with improved mechanical properties. It presents a glass transition temperature (Tg) of 53.7 °C. With a low density of 1.03 g/cm^3^ and rapid cooling and solidification rates, it enables precise layer placement without warping, maintaining dimensional accuracy. Its low hygroscopicity minimizes moisture absorption, reducing defects like bubbling or poor layer adhesion in open-air printing. Post-annealing, CFC PA exhibits a high tensile strength of 58 MPa and a Young’s Modulus of 1442 MPa, making it suitable for structural components requiring stiffness and flexibility. It also has a heat deflection temperature of 104 °C at 0.45 MPa, allowing it to perform well under elevated temperatures. Compared to standard polyamides like PA6 or PA66, CFC PA offers better processing ease and performance advantages, making it ideal for high-performance applications in the aerospace, automotive, and engineering sectors. While not biodegradable, its durability contributes to sustainability by reducing the need for frequent part replacements.

As reinforcement, continuous carbon fiber (CCF) produced by Anisoprint was utilized in this study. CCF is a carbon–epoxy composite composed of a bundle of 1500 carbon fibers, each with an average diameter of 7 μm, bonded together with an epoxy-based thermoset resin. According to the technical datasheet, CCF possesses an elastic modulus of 150 GPa, an ultimate tensile strength of 2200 MPa, and a fiber volume fraction of 60%. The printing process, which combines both the composite fiber co-extrusion polyamide and the CCF, results in a dual-matrix composite material. This composite presents a fiber volume fraction of 40%. This dual-matrix structure is formed due to the presence of both the thermoplastic matrix from the polyamide and the thermosetting resin from the carbon fiber, offering unique mechanical properties. The process parameters used to print the structures were derived by a previous work by the authors [25]. They are (i) macrolayer height of 0.32 mm, (ii) extrusion width of 0.65 mm, (iii) extruder temperature of 250 °C, and (iv) build plate temperature of 60 °C. 

Short carbon fiber (SCF)-reinforced polyamide was also used to 3D print reference isogrid structures. The PA filament is filled with 15% carbon fibers, and it was used in an FDM process. According to the technical datasheet, its tensile strength and tensile modulus are equal to 103 MPa and 8.38 GPa, respectively.

### 2.2. Compression Test

Compression tests were conducted on 3D-printed continuous fiber bimatrix composite specimens to evaluate the compressive mechanical behavior of the material. The test was conducted according to ASTM D6641, and the specimens were made with a length of 140 mm, thickness of 3 mm, and width of 13 mm. The gage length of the specimen was 13 mm. The specimens were printed with the fibers arranged in the direction of application of the compressive load. To ensure the repeatability of the test, five specimens were tested. A universal testing machine equipped with a 25 KN load cell was used to perform the tests. According to the standard, the appropriate test equipment was employed with a nominal rate of 1.3 mm/min. 

### 2.3. Isogrid Structures

**Figure 2:**
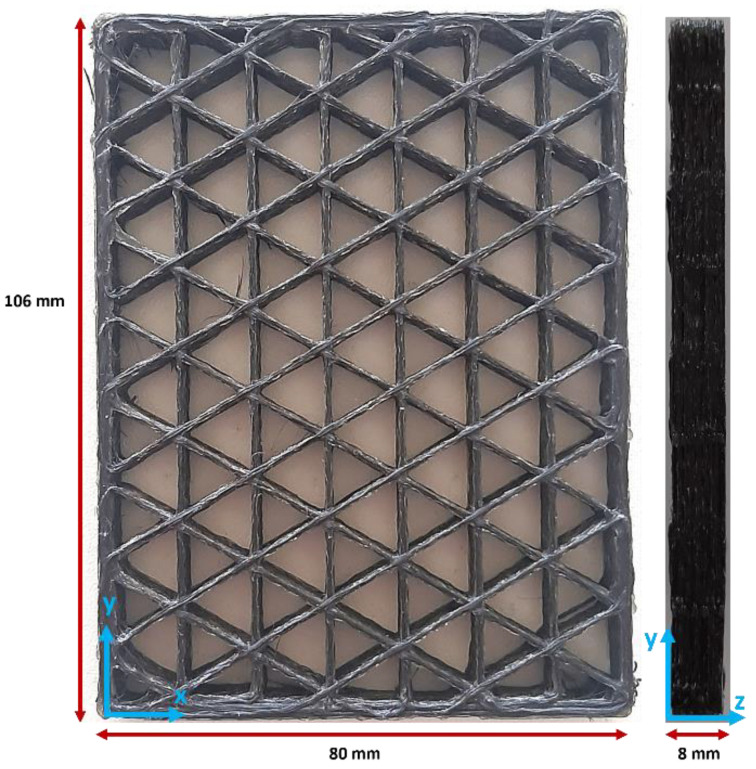
Example of the 30% isogrid.

Isogrid structures were created with CAD software Autodesk Inventor Professional 2024, originating from a parallelepiped with dimensions of 106 × 80 × 8 mm (Figure 2). This geometry was imported into the proprietary Anisoprint slicing software called Aura v. 2.4.8, where the Isogrid infill pattern was set, enabling the desired lattice structure to be achieved. In addition, the following printing parameters were set: printing speed of 6 mm/s, microlayer height of 0.32 mm, extrusion width of 0.65 mm, and build plate temperature of 60 °C. These parameters are the ones suggested by Anisoprint for achieving the best printing results. The dual-matrix composite filament extrusion was performed at a temperature of 250 °C. The isogrids were 3D printed at the following infill percentages: 10%, 20%, 30%, 40%, 50%, 60%, 70%, and 80%. To increase the accuracy of the tests, three isogrids were printed for each infill percentage.

**Figure 3:**
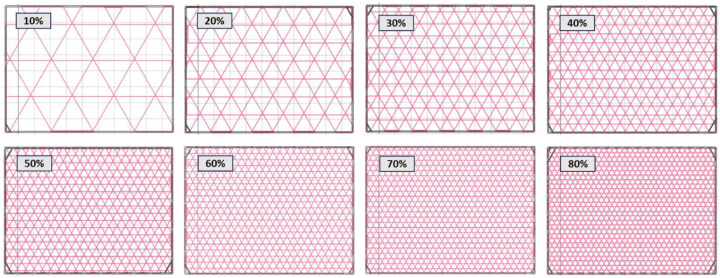
Infill densities of the isogrid structures.

Figure 3 illustrates the isogrid geometries at various infill densities. In the figure, the grey lines represent the reinforced perimeter of the structures, providing external support and stability, while the red lines indicate the reinforced infill, which contributes to the internal strength and buckling resistance of the isogrid configurations. These color-coded lines help visualize the distribution and arrangement of reinforcement within the isogrid structures, highlighting the differences in infill density and their potential impact on mechanical performance.

The reference isogrid structures in SCF were printed at 10% and 80% infill densities.

Before testing, the isogrid structures were weighted to determine their mass. The average values obtained from these measurements are presented in Table 1, both for continuous and short fiber-reinforced structures. These weight measurements are crucial for calculating the specific compressive resistance of the structures, allowing for a better understanding of the relationship between mass and mechanical performance.

### 2.4. Tomography Analysis

The 3D-printed lattice structures were analyzed using the ZEISS METROTOM 1500 industrial tomograph, a device that allows for high-resolution three-dimensional imaging. This technology utilizes X-rays to penetrate the object, providing detailed information about the internal structure of the isogrid, including porosity, defects, and complex or hidden geometries that are not visible through surface inspection alone. For the test, the isogrid structures were positioned with their shorter side on a metal support and placed on the rotating plate of the machine, located between the X-ray source and the X-ray detector within the machine chamber. The characteristic parameters of the testing machine are detailed in Table 2. This setup enabled precise imaging and analysis of the internal features of the 3D-printed structures, contributing to a deeper understanding of their material integrity and performance.

### 2.5. Buckling Test

**Figure 4:**
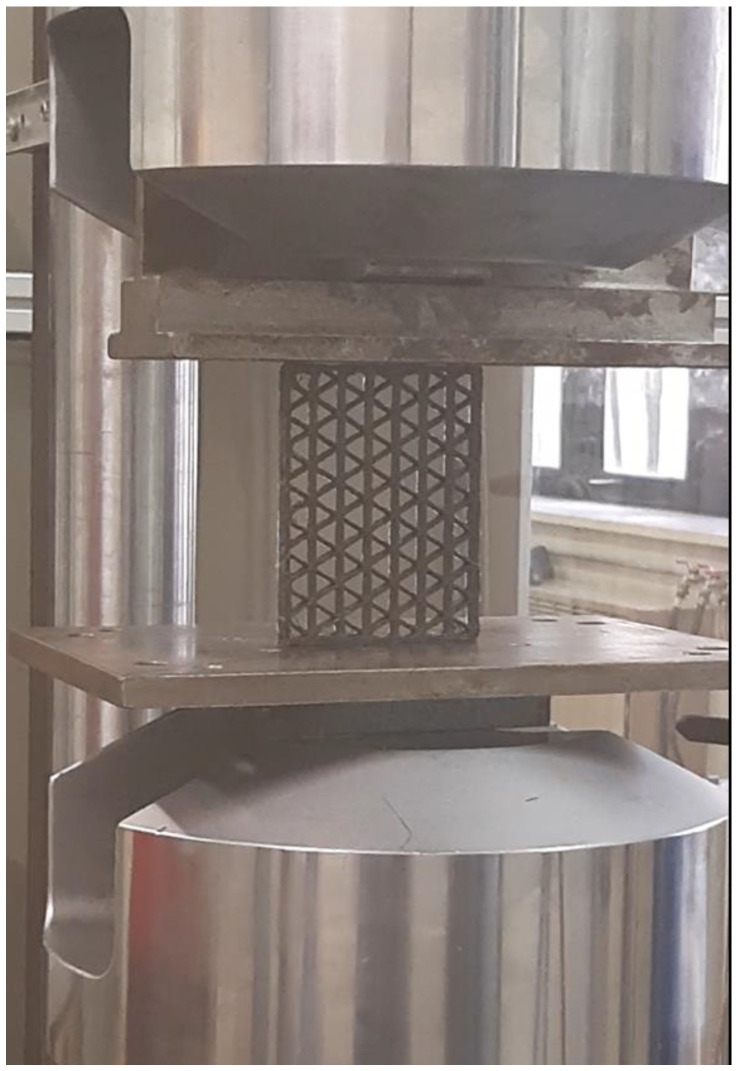
Buckling test of the 30% isogrid.

To examine the behavior of 3D-printed isogrid structures made from carbon fiber-reinforced polyamide, buckling tests were conducted using an MTS 810 servo-hydraulic testing machine, with the mobile platen moving at a speed of 0.5 mm/min (as shown in Figure 4). The isogrid structures were positioned such that their shortest sides were in contact with the machine plates, allowing the compressive load to be applied parallel to the fiber orientation, which is critical for accurately assessing the material buckling behavior. During the tests, a load cell and an inductive displacement transducer (LVDT) were used to record the values of load the maximum load (Pmax) and displacement (Δh) throughout the testing process. Additionally, the maximum specific buckling load (Pmax/w) was calculated by dividing the maximum buckling load by the weight of the lattice structure. This provides a normalized measure of buckling resistance relative to the structure mass.

Images capturing the failure mechanisms of the structures at different infill percentages were taken using a specialized camera, allowing for a detailed visual analysis of how varying infill densities affect the structural integrity under compressive loading.

### 2.6. Scanning Electron Microscope and Optical Analyses 

The examination of the isogrid morphology after the buckling tests was conducted using a Leica DMI8 optical microscope, provided by Leica Microsystems GmbH in Wetzlar, Germany. To gain more detailed insights into the fractured surfaces of the tested specimens, the field emission scanning electron microscope (FESEM) ZEISS SUPRA™ 40, equipped with a high-resolution GEMINI^®^ lens, was employed. This advanced microscope enabled the capture of finely detailed images, revealing the microstructural characteristics and failure mechanisms of the material. Prior to conducting the SEM analysis, a metallization process was applied to the specimens to enhance their electrical conductivity, ensuring the acquisition of high-quality electron microscope images and facilitating accurate analysis of the material’s internal structure.

## 3. Results

### 3.1. Compression Test

**Figure 5:**
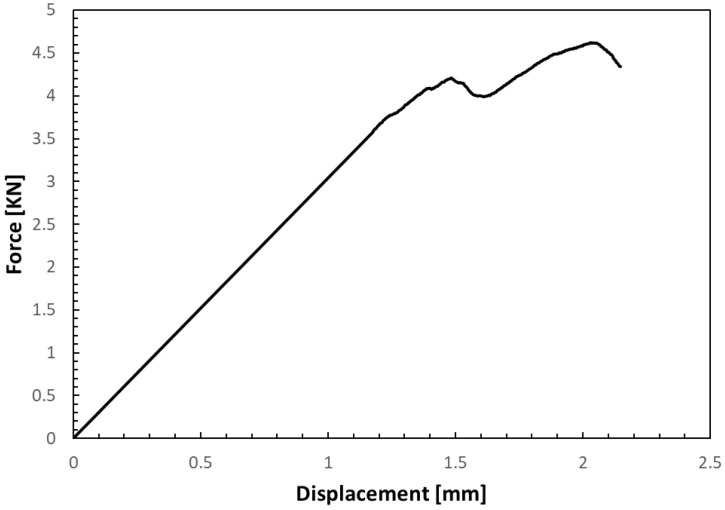
Typical compressive force–displacement curve.

Figure 5 illustrates the typical force–displacement curves that describe the compressive behavior of dual-matrix continuous carbon fiber specimens, which were 3D printed using the co-extrusion technique. The curve exhibits an irregular pattern due to the progressive failure of the composite material, which occurs with the formation of bend bands during the test [26]. As observed, once the elastic region is surpassed, the curve not only becomes more irregular but also displays a noticeable first peak around 4 KN. This initial peak corresponds to the onset of partial fiber delamination within the composite. Following this, a second peak appears at the point of fracture, marking the final failure of the specimen. The average maximum force recorded during the tests was 4.62 ± 0.43 kN, while the ultimate compressive strength of the specimens was 81.77 ± 1.50 MPa. These values highlight the material performance under compressive loading, with the irregularities in the curve providing insights into the complex failure mechanisms at play during the deformation and eventual failure of the 3D-printed composite.

### 3.2. Tomography Analysis

**Figure 6:**
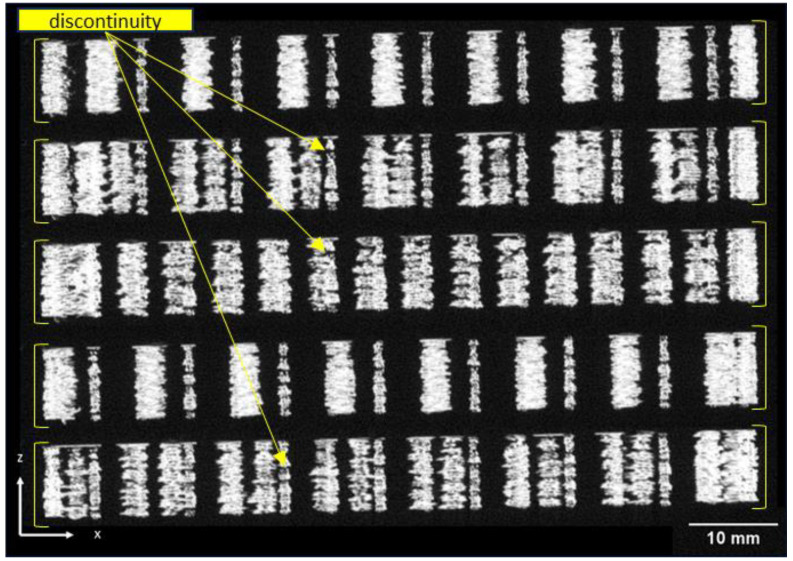
Tomography analysis of the 40% isogrid structure.

Figure 6 sequentially presents the tomography of five layers in the xz plane of the isogrid structure with 40% infill. The image highlights the emergence of macro-patterns at the intersections where the filaments are deposited. Specifically, at the nodes where filaments intersect, there is an overlap of dual filaments per layer, unlike along the ribs, where only single filaments are present. This overlap causes the filament to elevate slightly above the underlying layer as the deposition transitions from the rib to the node, creating areas of discontinuity within the structure. These discontinuity zones, which can potentially weaken the overall structural integrity, are located at different positions along the *y*-axis, as illustrated in the image. This observation is critical for understanding the internal structural dynamics and potential failure points within 3D-printed isogrid configurations.

### 3.3. Buckling Tests

**Figure 7:**
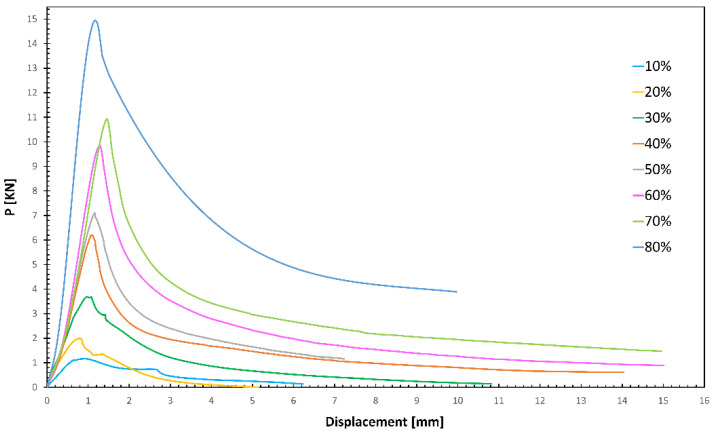
Average load–displacement curves.

Figure 7 shows the average load–displacement curves obtained by the buckling test of isogrid structures at different infills. The analysis shows that the buckling load Pmax (maximum load supported by the structures) always increases as the percentage of infill increases. It can be observed that the curves corresponding to low infill percentages (10%, 20%, and 30%) show a similar behavior. In fact, after reaching the peak load, the curves show a step that becomes less evident as the infill density increases. The initial peak corresponds to the beginning of local buckling in the vertical ribs, while the subsequent peak is due to the onset of global buckling, caused by a greater displacement. In contrast, isogrids between 40% and 80% infill present a single peak corresponding to the onset of global buckling.

Table 3 presents the average values of the buckling load and specific buckling load achieved for different infill densities, along with their respective standard deviations. Moreover, the results of the buckling test on the SCF-reinforced isogrid are reported as a reference. The data indicate that, similar to the trend observed with the maximum buckling load, the maximum specific buckling load also increases as the infill percentage increases. This relationship underscores the importance of infill density in enhancing the structural integrity and buckling resistance of the 3D-printed isogrid structures. The standard deviations provide an indication of the variability in the measurements, highlighting the consistency of the results across different samples. At 80% infill, the CCF structure shows an increase of 66% and 61.5% in maximum load and maximum specific load, respectively.

The comparison with SCF indicates that the use of CFC significantly contributes to the buckling resistance, as the increases in the maximum specific buckling load for the 10% and for the 80% infill are equal to 16 and 85%, respectively.

**Figure 8:**
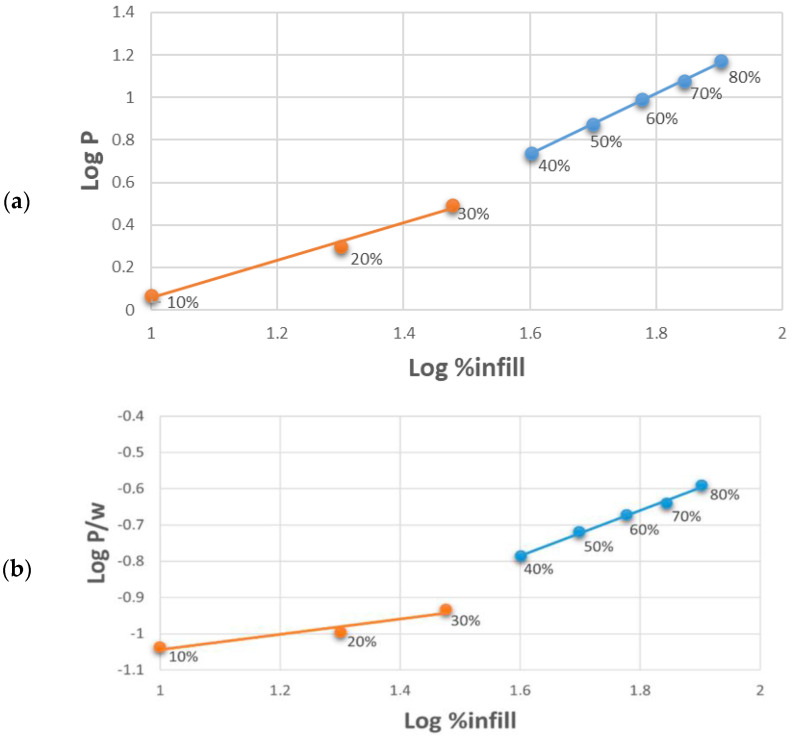
Buckling load (**a**) and specific buckling load (**b**) as a function of % infill, reported on a logarithmic scale: emphasis on the change in slope between lower 30% infills and higher.

The graphs in Figure 8 represent the trend, on a bi-logarithmic scale, of the buckling load (a) and of the specific buckling load (b) as the infill percentage varies. The rate of growth of the buckling load as a function of the infill % is lower at low percentages (up to 30%) and then increases at high percentages; in fact, two different slopes of the curve are evident. The change in slope becomes more pronounced when considering the specific buckling load, with an increase in the angular coefficient of the straight line, describing the behavior of the load as the infill density increases by 66% when going from 30% to 40%.

**Figure 9:**
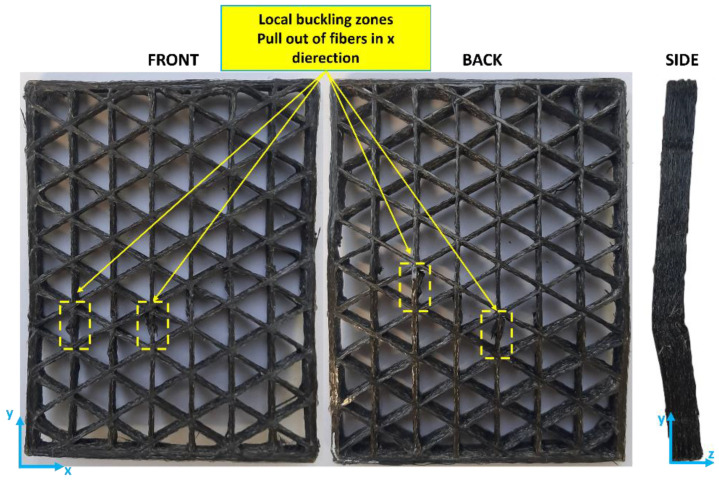
The tested 30% infill isogrid structure.

The distinct behavior of structures with infill densities of up to 30% compared to those with higher densities can be attributed to the different buckling mechanisms these structures undergo. Specifically, structures with 10%, 20%, and 30% infill densities tend to fail through local buckling upon reaching the critical load. In these cases, failure is confined to specific zones within the structure, typically occurring on the xy plane, as illustrated in Figure 9. Conversely, at higher infill densities, the failure mechanism involves the entire structure, leading to global buckling. This global failure is characterized by deformation and buckling that extends across the entire cross-section of the isogrid. 

**Figure 10:**
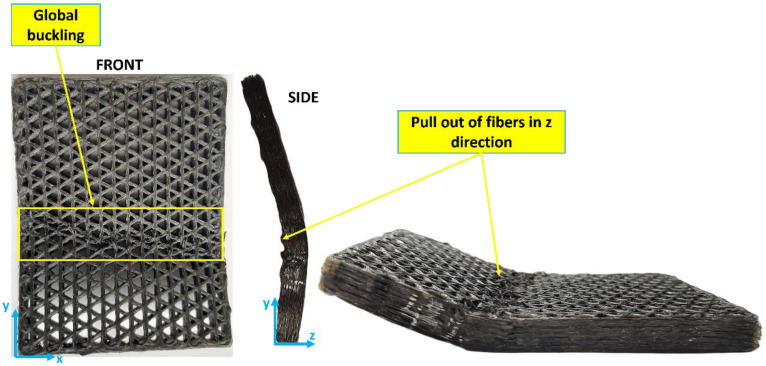
The tested 60% infill isogrid structure.

Visual inspection of the isogrids after failure further supports these observations. Structures with up to 30% infill show localized failure zones, whereas those with higher infill densities exhibit more widespread failure, involving the entire structure. Additionally, in the higher-density structures, fiber pull-out is observed not only on the xy plane but also in the z direction, indicating a more complex and extensive failure mechanism, as shown in Figure 10. These findings highlight the critical role of infill density in determining the buckling behavior and overall structural integrity of 3D-printed isogrid configurations.

### 3.4. Optical and Scanning Electron Microscope Analyses

**Figure 11:**
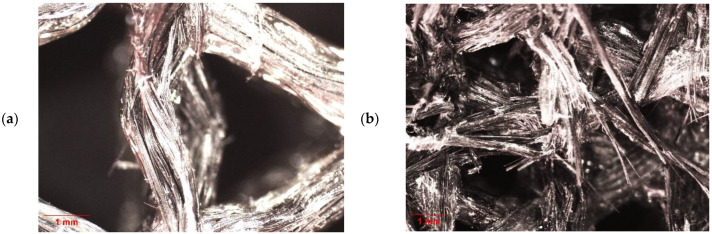
(**a**) Infill 30%, 16×, local buckling; (**b**) infill 60%, 12.5× global buckling.

The morphology of the isogrids after the buckling tests was observed through an optical microscope. Figure 11 shows the 16× and 12.5× magnifications of the isogrids at 30% and 60% infill. Comparison of the two images highlights distinct failure mechanisms, which are related to the different modes through which the two structures reach buckling, either local or global. Indeed, the optical micrograph of the 30% isogrid shows a failure of the fibers on the xy plane (also observable in the 10% and 20% structures), while the 60% isogrid exhibits a different failure mechanism, with most of the fibers failing on the xz and yz planes.

**Figure 12:**
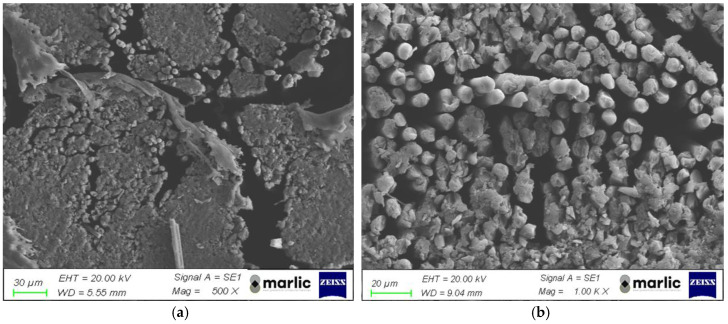
(**a**) The 500× and (**b**) 1000× SEM magnification of the 3D-printed composite.

The image of the fracture surfaces of the 3D-printed lattice structure was also observed by exploiting SEM (Figure 12). The presence of macrovoids between the deposited fiber bundles is evident (recognizable at 500× magnification), which leads to a non-homogeneous structure. Single carbon fibers can also be noted. On the other hand, the 1000× magnification highlights the presence of microvoids between the single filaments. The presence of macro- and microvoids can be attributed to deposition issues and low and inhomogeneous impregnation of the fiber during extrusion. These defects also affect the mechanical properties of the material itself. Moreover, they compromise the material mechanical properties and make it difficult to achieve the expected mechanical performances. Furthermore, the voids significantly impact the material’s ability to absorb humidity, further affecting its overall integrity and functionality.

## 4. Conclusions

In this study, composite isogrid structures made from continuous carbon fiber-reinforced polyamide were fabricated using a 3D-printing process. The research focused on analyzing the effect of varying infill densities on the buckling behavior of these isogrid structures. The key findings from this investigation are summarized as follows:

- The compressive behavior of the 3D-printed specimens closely resembles that of traditional composite materials, demonstrating comparable mechanical responses under load.
- During the buckling tests, the load applied to the isogrid structures increases with displacement, reaching a peak value that corresponds to the onset of buckling. This behavior is consistent with theoretical predictions for buckling phenomena.
- The failure modes of the isogrid structures are influenced by the infill density. Structures with lower infill density predominantly fail through local buckling, characterized by deformation confined to specific areas. Conversely, structures with higher infill density tend to fail through global buckling, where the entire structure deforms uniformly.
- The maximum buckling load increases as the lattice infill density increases, regardless of the type of buckling failure. This indicates that denser structures are better able to withstand compressive forces before failing.
- The rate at which the buckling load increases is modest at lower infill densities but becomes significantly steeper at higher densities. This suggests that there is a threshold density beyond which the structural integrity is markedly enhanced.
- The specific buckling load, which normalizes buckling resistance against the structure’s weight, shows a trend similar to that of the absolute buckling load. This implies that as the structure’s weight increases, so does its resistance to buckling, making heavier but denser structures more robust.
- Continuous carbon fibers significantly increase both the maximum buckling load and the maximum specific buckling load compared to short carbon fibers.
- Microscopy analyses provide insights into the failure mechanisms, clearly distinguishing between local and global buckling modes in the lattice structures. These observations are crucial for understanding how to design against specific types of failure.
- Scanning electron microscopy images reveal the presence of both macro- and microvoids within the composite structure. These voids are critical defects that negatively impact mechanical performance, indicating areas for potential process improvements.

This research aims at demonstrating that specific strength can still be improved with increased material usage, even when different buckling modes occur. These findings are critical for the design of stronger and lighter 3D-printed composite structures, particularly in industries like aerospace and automotive engineering, where both weight reduction and strength are essential.

Isogrid lattice structures manufactured from composites via 3D printing exhibit high buckling loads, particularly when local buckling is minimized. However, the presence of voids remains a significant challenge, underscoring the need for further process optimization to reduce void formation and enhance structural performance. Future research will focus on reducing the porosity and the void quantity in the structures by optimizing 3D-printing parameters and by performing post-processing operations in order to produce more efficient components, and on exploring the impact of moisture absorption on the buckling resistance of these isogrid structures.

## Figures and Tables

**Table 1:** Average weight of isogrid structures.

% Infill	10		20	30	40	50	60	70	80	
	CCF	SCF	CCF	CCF	CCF	CCF	CCF	CCF	CCF	SCF
Weight [g]	12.72	10.82	19.73	26.79	33.54	39.04	46.12	52.19	58.23	51.2
Standard deviation	±0.5	±0.9	±1.2	±0.6	±0.8	±1.3	±0.7	±0.4	±0.6	±0.8

**Table 2:** Tomograph settings used for the isogrid structure analysis.

Voltage	180 KW
Current strength	380 μA
Voxel	64.08 μm
Volume	303 × 1479 × 1824 Voxel
Filter	Cu 0.5 mm
Integration time	666 ms
X-ray beam size	68 μm

**Table 3:** Average maximum load and maximum specific load as the % infill changes.

% Infill	10		20	30	40	50	60	70	80	
	CCF	SCF	CCF	CCF	CCF	CCF	CCF	CCF	CCF	SCF
Maximum load (Pmax) [KN]	1.16 ± 0.12	0.85 ± 0.12	1.99 ± 0.23	3.12 ± 0.35	5.49 ± 0.32	7.45 ± 0.15	9.80 ± 0.24	11.93 ± 0.17	14.94 ± 0.20	7.08 ± 0.30
Maximum specific buckling load (Pmax/w) [KN/g]	0.091 ± 0.009	0.078 ± 0.016	0.101 ± 0.012	0.117 ± 0.013	0.164 ± 0.010	0.191 ± 0.004	0.212 ± 0.005	0.229 ± 0.003	0.257 ± 0.004	0.138 ± 0.005

## Data Availability

The original contributions presented in the study are included in the article, further inquiries can be directed to the corresponding author.
